# Shining a light on emerging talent: introducing ‘First Authors in the Spotlight’

**DOI:** 10.1007/s12471-024-01884-6

**Published:** 2024-07-11

**Authors:** Pim van der Harst

**Affiliations:** Department of Cardiology, Division of Heart & Lungs, University Medical Center Utrecht, Utrecht, The Netherlands

Recently, we introduced the ‘Letters to the Editor’ series to initiate a discussion between our readers and authors [1]. In this edition, the discussion focuses on aspects of recently published work on the transcatheter closure of postsurgical aortic pseudoaneurysms [2], leading to further explanation of key points and a corrigendum regarding the exact percentage referred to by the authors [3].

I am now pleased to introduce another new feature in the Netherlands Heart Journal: ‘First Authors in the Spotlight’. This series aims to provide insights into the journeys, motivations, contributions to cardiovascular medicine, and personal interests of the first authors of our original articles. In this edition, we spotlight the first authors of four original articles. These researchers report new developments in their fields, from TAVI studies to pioneering treatments for ventricular tachycardia and improving outcomes in surgery and cardiogenic shock. Their work, performed in Zwolle, Nijmegen, Twente and Eindhoven, offers insights into the progress of cardiovascular disease treatment throughout the Netherlands.

The ‘First Authors in the Spotlight’ series aligns with our goal to increase visibility, and recognise and support young talents and emerging leaders in cardiovascular research [4]. By highlighting these individuals, we aim to inspire our readers with stories of dedication and innovation. By providing a face and a story, we hope to facilitate their recognition within the Netherlands Society of Cardiology (NVVC) community, making it easier for them to connect with colleagues and potentially open up discussions on their work.

We hope you find this new series as interesting and informative as we do, and that it sparks further discussion. We look forward to sharing more stories of our first authors in future issues.
